# Violent paroxysmal activity drives self-feeding magma replenishment at Mt. Etna

**DOI:** 10.1038/s41598-019-43211-9

**Published:** 2019-04-30

**Authors:** Marco Viccaro, Marisa Giuffrida, Francesco Zuccarello, Mariabenedetta Scandura, Mimmo Palano, Stefano Gresta

**Affiliations:** 1 Università di Catania, Dipartimento di Scienze Biologiche, Geologiche e Ambientali - Sezione di Scienze della Terra, Corso Italia 57, I-95129 Catania, Italy; 2 Istituto Nazionale di Geofisica e Vulcanologia, Sezione di Catania - Osservatorio Etneo, Piazza Roma 2, 95125 Catania, Italy

**Keywords:** Geophysics, Volcanology

## Abstract

A new sequence of eruptions occurred at Mt. Etna volcano during the first half of 2017, after almost 8 months of quiescence. These episodes had low-to-mild intensity and markedly differ from the violent paroxysms occurred at the Voragine Crater (VOR) during December 2015 and May 2016. Despite the general weak explosive nature of the eruptions, the activity during 2017 revealed unusually complex dynamics of magma ascent and interaction. Detection and investigation of such dynamics required a multidisciplinary approach in which bulk rock compositions, crystal chemical zoning, diffusion chronometry and ground deformation data have been combined. Bulk rock major and trace elements suggest that the 2017 magmas followed a differentiation path similar to that experienced by magmas erupted at Mt. Etna during the 2015–16 eruptions at VOR. Olivine core compositions and zoning patterns indicate the presence of multiple magmatic environments at depth that strictly interacted each other through some episodes of intrusion and mixing before and during the 2017 eruptive events. Timescales retrieved from diffusion chronometry on olivine normal and reverse zoning correlate well with the ground deformation stages detected through geodetic data and associated models, thus allowing to track the evolution through time of the 2017 volcanic activity. Combination of all petrological and geodetic observations supports the idea that dynamics of magma transfer driving the eruptive episodes of 2017 have been a direct consequence of the violent eruptions occurred at VOR on May 2016, which boosted the ascent of new magma from depth and improved the efficiency of the plumbing system to transfer it upward to the surface. We propose a mechanism of self-feeding replenishment of the volcano plumbing system during 2017, where magma recharge from depth is triggered by sudden unloading of the magma column consequential to the violent paroxysmal activity occurred on May 2016 at VOR.

## Introduction

In the last few decades, several eruptions have taken place at Mt. Etna in relatively short succession, passing from periods of entirely effusive to strongly explosive activity (i.e., violent Strombolian to lava fountains). Since 2011, the eruptive behavior was dominantly explosive with two major cycles of paroxysmal eruptions as those of 2011–2013 at the New South East Crater (NSEC) and 2015–2016 at the Voragine Crater (VOR), which were separated by a weak explosive/effusive eruption occurred on the lower eastern flank of the North East Crater (NEC) and the NSEC on July-August 2014. During the first months of 2017, the activity turned to dominantly effusive, giving rise to a short sequence of weak Strombolian explosions and lava flow emissions between February and April. Understanding and forecast such eruptive phenomena are of prominent importance and of considerable impact especially in those volcanic areas characterized by high population density. In order to enable a better knowledge of the volcanic phenomena in progress at Mt. Etna, the observation and monitoring systems have been significantly upgraded in recent years, and a higher quantity and quality of instrumental and analytical data associated to recent eruptions have been collected^1–3^. In particular, the rapid growth of continuously recording geodetic networks enhanced the acquisition of extensive datasets that largely improved our current knowledge of the magmatic plumbing system, providing constraints on subsurface movements of fluids (i.e., magma, volatiles or hydrothermal waters)^4^ as well as the geometry, size, and depth range of magmatic sources^4^. However, in the case of articulated plumbing systems, geodetic data modeling alone is unable to deconvolve complex evolutionary dynamics of magmas, comprehensively track the ascent paths of magmas, and potentially resolve their temporal relationships without relying on compositional information preserved in volcanic minerals and rocks.

In this study, we show that geochemistry and diffusion chronometry applied to compositionally-zoned crystals may provide the fundamental framework for the interpretation of geodetic data associated to complex volcanic phenomena. Based on an extensive dataset of concentration and diffusion profiles of olivine crystals combined with geodetic observations, we are able to interpret the volcanic unrest throughout the 2017 eruptive period in terms of deep and subsurface pre-eruptive processes such as the storage, transport and interactions of magmas. Such approach of investigation allows the spatial localization of active magmatic sources, and also defines their temporal activation before and during each eruptive episode. This enables to address some important changes in the modes of magma supply into the Etnean plumbing system during 2017, which is a direct consequence of the last violent paroxysmal episodes occurred at VOR on May 2016.

## Volcanological Background

**Figure 1:**
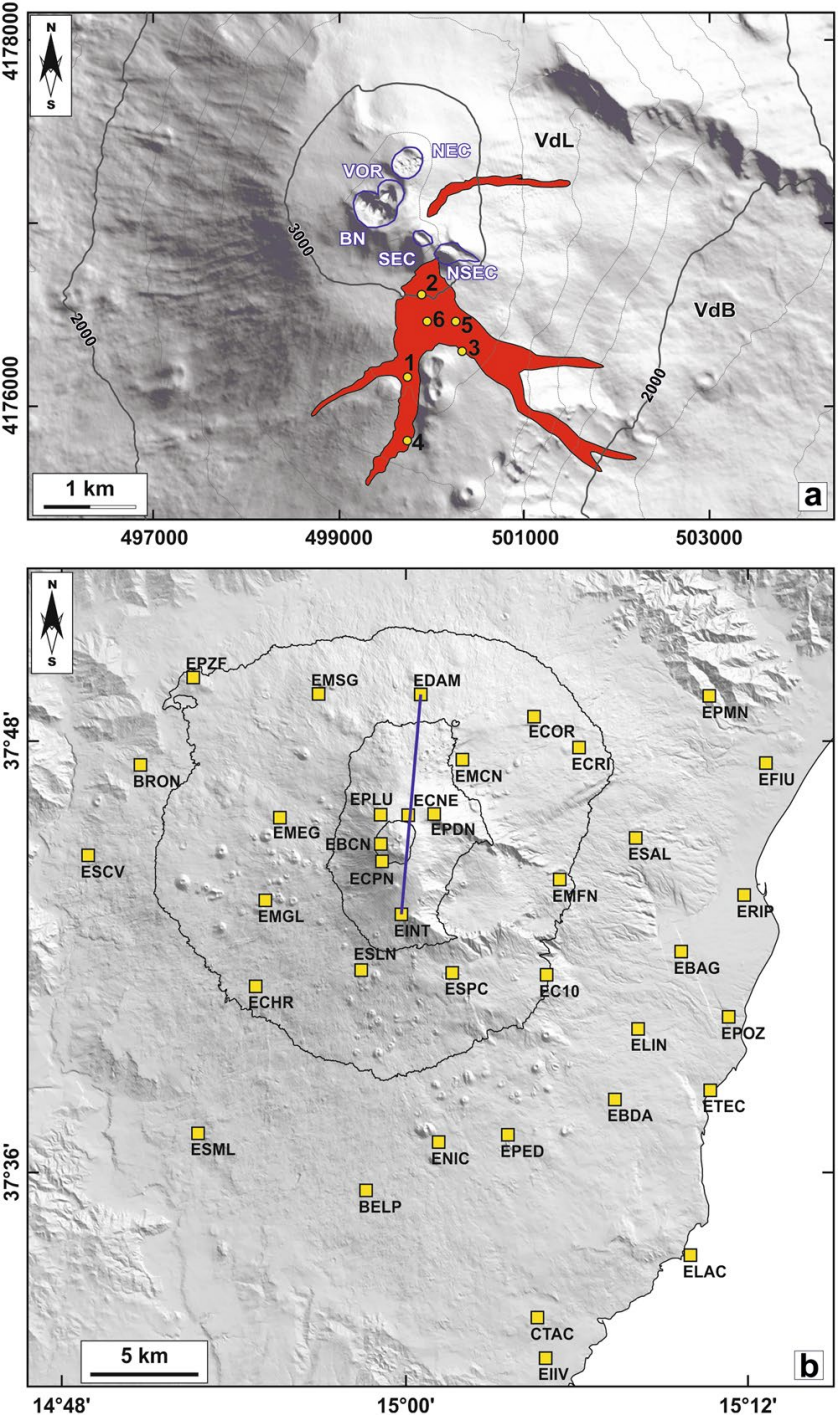
(**a**) Digital elevation model of the summit area of Mt. Etna volcano with the five summit craters: Bocca Nuova (BN); Voragine (VOR); North-East Crater (NEC); South East Crater (SEC); New South East Crater (NSEC). The lava flow field related to the February–April 2017 volcanic activity is also reported (Valle del Bove, VdB; Valle del Leone, VdL). Numbers refer to the location of collected samples: (1–2) lava samples collected on February 28; (3) samples collected during the eruption of March 19; (4) samples collected on March 25; (5) samples of April 11; (6) samples collected on April 19. (**b**) Topographic map of Mt. Etna with location of the GPS stations used in this study (the EDAM-EINT baseline is traced with a blue line).

Volcanic activity resumed at Mt. Etna after almost 8 months of quiescence since the last eruptive episodes occurred at VOR in May 2016. Ash emission and a modest Strombolian activity started at a vent located on the saddle between South East Crater (SEC) and NSEC since January 20, 2017 (Fig. 1a). The explosive activity became more vigorous during the night between February 27 and 28, with lava flow emissions from the southern flank of the SEC until March 1, when both explosive and effusive activity ceased temporary. After two weeks of ash emission from the vent at the SEC-NSEC saddle, Strombolian activity resumed in the morning of March 15. Such activity was also accompanied by lava flow emission directed to south, covering flows of the February 27–28 activity (Fig. 1a). A change in eruptive dynamics occurred during the early hours of March 16. Indeed, a fracture opened at the base of the southern flank of SEC and started to feed effusive activity. This lava flow moved to southeast, reaching the western border of Valle del Bove in the morning of the same day, while the activity at SEC was characterized by conspicuous ash emission (Fig. 1a). During this eruptive phase, various explosions occurred in the front of the lava flow due to interaction between lava and the snow coverage. Although the explosive activity at SEC ended on March 19, the effusive activity from the fracture at the base of SEC continued to feed lava flows toward south-southwest until April 9. The effusive activity resumed again on April 10 from the same fracture. Other weak Strombolian eruptions took place on April 13–15 and April 19–21 at the vent located on the SEC-NSEC saddle, and were accompanied by emission of new lava flows from fractures located on the southern flank of the SEC. These flows were directed to south and southeast toward the Valle del Bove and reached an altitude of 1950 m asl (Fig. 1a). During April 26–27, a new Strombolian eruption coupled with lava flow effusion occurred at the SEC-NSEC saddle. Simultaneously, two fractures on the northern flank of SEC fed a new lava flow that propagated toward the Valle del Leone (Fig. 1a). On May 2017, SEC was characterized by episodic intra-crateric activity that completely ceased during June 2017.

## Petrography and Geochemistry of the Erupted Products

Twelve lava samples were taken at the base of the SEC-NSEC cones at Mt. Etna during the emplacement of lava flows related to the most significant eruptive episodes encompassing the activity of February–April 2017 (Fig. 1a). The collected samples were immediately quenched to prevent post-emplacement diffusion. The investigated volcanic rocks are lavas with porphyritic structure and phenocryst volume ranging between 20% and 30%. The mineral assemblage is constituted by phenocrysts of plagioclase (50–60 vol.% of the total phenocryst abundance), augitic clinopyroxene (20–35 vol.%), olivine (5–10 vol.%) and opaque oxide (5 vol.%). The groundmass is dominantly hyalopilitic, with microlites of plagioclase, augitic clinopyroxene, opaque oxides and subordinate olivine. Lava samples of March 25 and April 11 locally show portions characterized by scarce mafic minerals (i.e., olivine and clinopyroxene) testifying episodes of magma mingling. The petrology of all samples along with textures of phenocrysts are very similar to those already observed in products of the historic and recent eruptions of Mt. Etna^5–11^, with a much closer resemblance to products of the post-2011 activity^6,7,10,11^. Phenocrysts show large variability for what concerns their sizes, textures and compositions. Plagioclase (An_51–88_) is euhedral to subhedral, with sizes variable from 100 µm (micro-phenocrysts) up to 4 mm. Cores and rims of most of plagioclase crystals are commonly affected by a variety of disequilibrium textures (i.e., coarse sieve-textures, dissolution/resorption textures), which superimpose to oscillatory zoning patterns. As a whole, most of crystals exhibit cores with coarse sieve-textures and rims with sieve-textures or oscillatory zoning, whereas totally oscillatory-zoned crystals are quite rare in all samples under investigation. Augitic clinopyroxene is found as individual crystals with size between 100 µm and 5 mm or as aggregates including olivine and opaque oxides. Mg# ranges between 0.82 and 0.72, with a major peak at 0.77–0.78. The largest crystals (>1 mm in size) are generally euhedral, whereas the subhedral habitus is typical of smaller-sized clinopyroxenes. Disequilibrium features similar to sieve-textures also characterize the rim of some individual crystals. Olivine crystals (Fo_65–81_) are euhedral to subhedral, and 200–500 µm in size. Few individual crystals exhibit larger sizes up to ~2.5 mm, and are often characterized by pervasive fractures and embayments. Olivines with resorbed rim constitute ~30% of the total observed olivine phenocrysts and are particularly common in lavas of the February and April 2017 eruptions. In particular, lava samples erupted during April 2017 show the most destabilized crystals with rims affected by pervasive spongy textures. Opaque oxides are anhedral titaniferous magnetites that are mostly enclosed in large crystals of olivine and augitic clinopyroxene. Compositions of the observed mineral phases are provided as Supplementary Tables S1 and S2.

**Figure 2:**
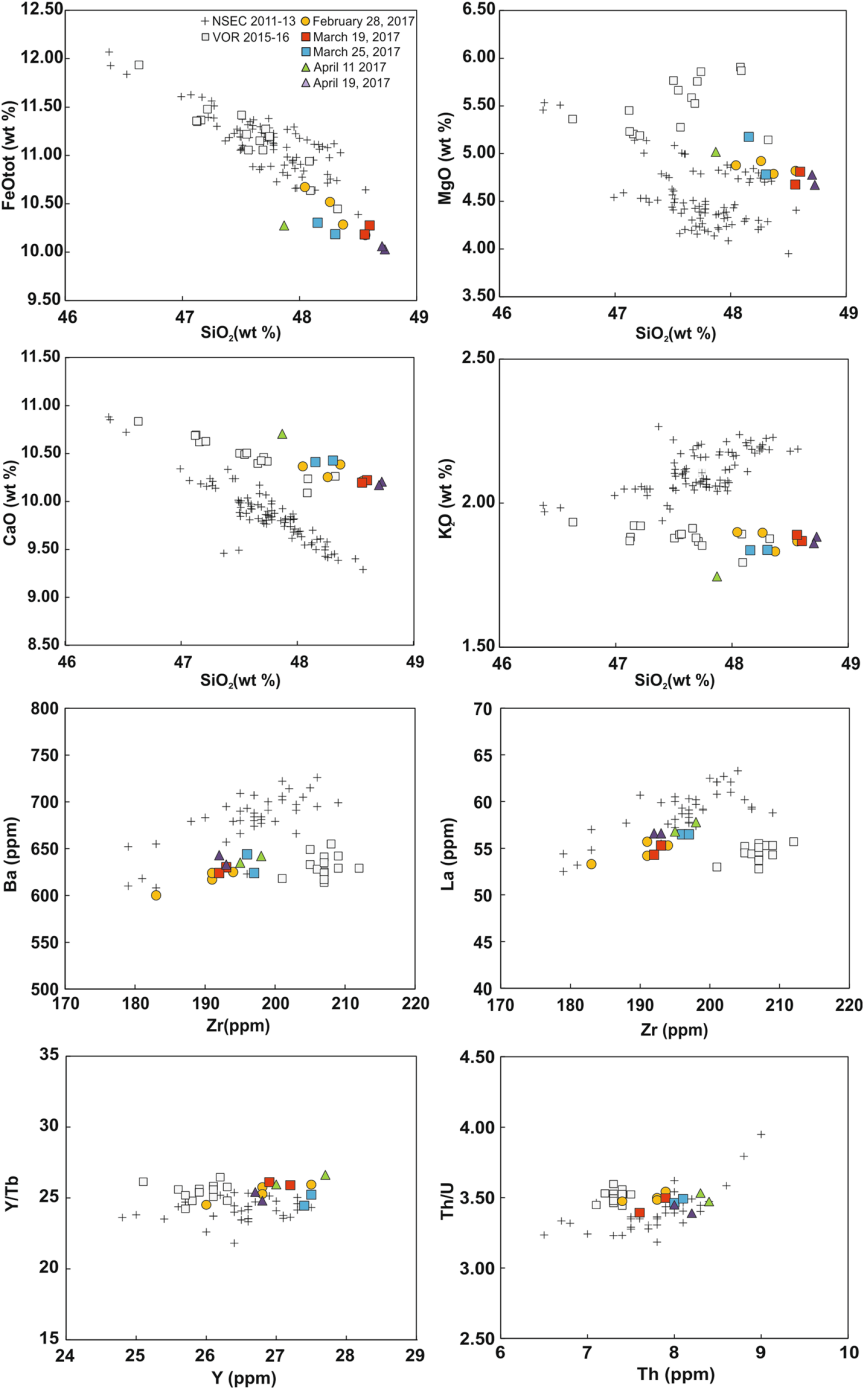
Diagrams showing the variation of selected major and trace elements plotted against SiO_2_ (wt.%) and Zr (ppm) respectively, together with selected incompatible trace element ratios for volcanic rocks erupted during the February–April 2017 activity. Compositions of the products erupted during the 2011–2013 activity at the NSEC^6^ and the 2015–2016 eruptions at VOR^11^ have been reported for comparison.

Volcanic rocks erupted during the activity of February–April 2017 at Mt. Etna are K-trachybasalts with major element compositions consistent, on the whole, with those of products emitted between 2011 and 2016 from NSEC, NEC and VOR (Supplementary Table S3)^6,7,10,11^. Within the whole 2011–2017 suite, major element abundances of the 2017 volcanic rocks are more akin to those of products emitted at VOR during 2015–2016 (Fig. 2). Indeed, the 2015–2017 products display common trends of evolution for what regards all the major elements, with slight discrepancies in some elements (chiefly CaO, MgO and K_2_O) with respect to the patterns defined by volcanic products erupted at the NSEC during 2011–2013 (Fig. 2). Trace element abundances of the February–April 2017 products confirm the picture resulting from major elements, being the Large Ion Lithophile Elements (LILE; Rb, Ba and Sr) and all the Rare Earth Element (REE) group very comparable with those of the VOR 2015–2016 volcanic rocks (Fig. 2; see also Supplementary Table S3). More complex is the variability among the High Field Strength Elements (HSFE): if compared to abundances of the VOR 2015–2016 products, Zr concentrations of the 2017 volcanic rocks are markedly lower, U, Th and Y are higher, whereas Nb is rather comparable (Fig. 2; see also Supplementary Table S3). Even in terms of incompatible trace element ratios, the 2017 lavas display geochemical characteristics more similar to those of the VOR 2015–2016 products (Fig. 2; see also Supplementary Table S3). The analyzed products of 2017 also display geochemical variations that can be correlated with the time of emission throughout the February–April eruptive episodes. Specifically, compositions become slightly more evolved (i.e., MgO decreases) from February 28 to March 19, whereas a tendency to more basic compositions is recorded for products erupted on March 25. MgO decreases again for products emitted during April 11, reaching concentrations in the April 19 volcanic rocks very close to those of February 28 and March 19 samples.

## Olivine Compositional Zoning

**Figure 3:**
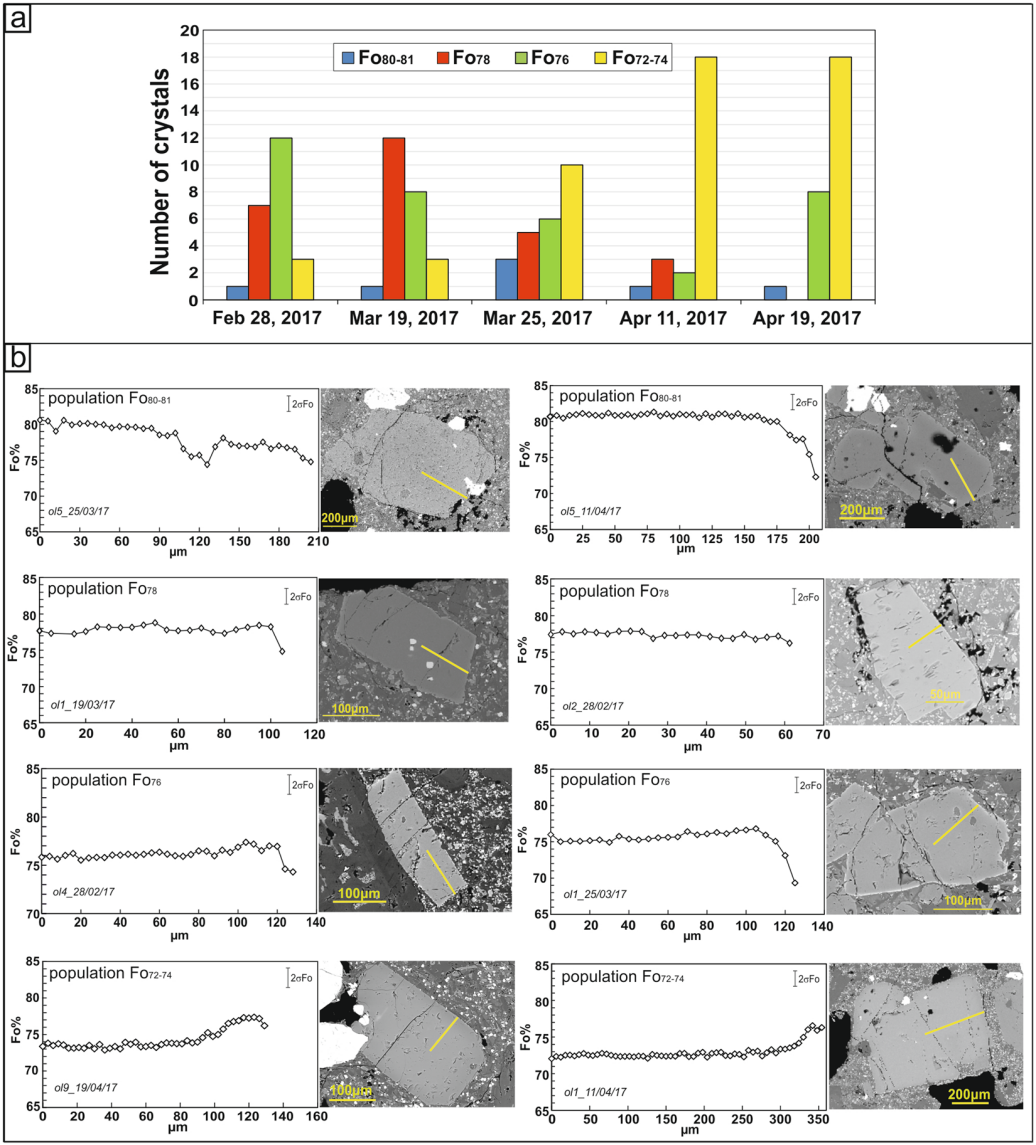
(**a**) Number of olivine crystals related to each population identified in the February–April 2017 volcanic rocks. (**b**) Core-to-rim compositional profiles in representative olivine crystals in each olivine population. Yellow lines in the BSE images indicate the direction of the SEM-EDS/WDS compositional traverses.

Major element compositions have been analyzed for a total of 122 olivines (Fig. 3a). Sixty-nine olivine crystals were then selected for measurement of major element concentrations along core-to-rim transects with spacing of 3–7 µm (see Supplementary Table S2). On the whole, olivine phenocrysts are in the size range of 200–600 µm and show forsterite contents varying between Fo_69_ and Fo_81_. No evident correlations are observable between crystal sizes and Fo compositions. For the eruptive episodes investigated in this study, we are able to distinguish different olivine zoning patterns varying from normal to reverse to more complex zoning types. Specifically, for the samples of February 28 and March 19, 2017 we observed that the majority of olivine crystals are characterized by rather flat core-to-rim chemical profiles or they have homogeneous core compositions and normally zoned rims. Starting from March 25, we observed a major frequency of reverse zoning patterns in olivine crystals and also the occurrence of more complex zoning (e.g., crystals interiors that are reversely zoned and rims normally zoned, or normally zoned interiors coupled with reverse zoning at the olivine rim; Supplementary Table S2). Reverse zoning becomes particularly common in olivine crystals erupted on April 11 and April 19, 2017. By taking into account the composition of the inner cores as well as the entire core-to-rim zoning profile, we have grouped the olivine crystals into populations that show compositional affinities with olivine populations previously identified in other products of the post-2011 activity of Mt. Etna^7,10,11^. The less evolved population has core composition at Fo_80–81_, which is represented by only few individual crystals that have been observed throughout the February–April period (Fig. 3a). The dominant zoning patterns characterizing such population is normal (Fig. 3b). Two crystals show more complex patterns with Fo_80–81_ cores surrounded by mantles with normal zoning (Fo concentration decreases to Fo_74_ or Fo_76_), then shifting to reverse zoning (Fo increases up to Fo_78_ or Fo_81_) and again to normal (decreasing concentration to Fo_72–75_) at the outermost rim (Fig. 3b). Olivine with cores at Fo_78_ is particularly abundant in products erupted between February and March 2017, with a peak in products of March 19 (Fig. 3a). Differently, Fo_78_ olivine cores decrease in products of April 11 and are totally lacking in those of April 19 (Fig. 3a). These crystals display rather flat core-to-rim profiles or they have zoned rims with concentrations decreasing from the crystal interior to Fo_73–75_ (Fig. 3b). We also found olivine crystals having core compositions at Fo_76_, which are rather homogenously distributed throughout the whole February–April 2017 period (Fig. 3a). All crystals with Fo_76_ cores have variegated zoning patterns, varying from flat or slight reverse profiles (up to Fo_77–78_) to normal zoning toward the crystal edges (Fo_73–74_; Fig. 3b). Over the period under investigation, we have found another olivine population represented by more differentiated cores at Fo_72–74_. In particular, cores with Fo_74_ compositions are the most abundant and characterize olivine crystals erupted from March 25 to April 19 (Fig. 3a). Fo_72–74_ cores are rather flat and mantled by regions with higher Fo contents (Fo_75–78_; Fig. 3b). Moreover, in some of these crystals, the reversely zoned regions (up to Fo_78_) are surrounded by normally zoned rims (Fo_72–76_).

## Geodetic Data

**Figure 4:**
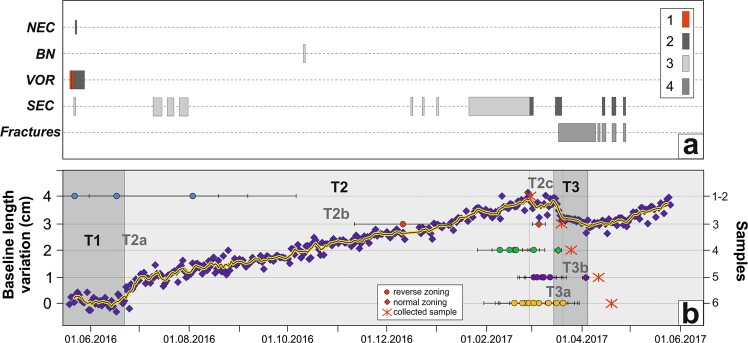
The panel (a) shows the temporal evolution of the volcanic activity at summit area: (1) fire fountain activity; (2) Strombolian activity; (3) ash emission/weak explosive activity; (4) effusive activity from the fracture. The panel (b) displays daily baseline changes of EDAM and EINT stations (see the map location in Fig. 1b). The visual inspection of such a baseline allows the detection of the main ground deformation stages defined as T1, T2 and T3. T2a, T2b, T3a and T3b sub-periods show marked differences with respect to the general T2 and T3 patterns. Timescales retrieved through diffusion modeling in zoned olivines from each sample have been reported for a direct comparison with ground deformation stages (see also Table 2 and Fig. 7 for the specific connections between the modeled magmatic environments). Numbers at the right y-axis of the panel (b) refer to sample locations as in Fig. 1a.

Raw GPS observations collected from the permanent network (Fig. 1b) and spanning the May 19, 2016–May 25, 2017 period were analyzed using the GAMIT/GLOBK software^12^ and adopting a methodology commonly used at Mt. Etna^13,14^. To detect significant changes associated with Mt. Etna activity, we inspected the daily baseline changes of a pair of stations (EDAM-EINT; Fig. 1b) that quasi-continuously operated throughout the investigated period (Fig. 4). Changes over time of this baseline are able to track magma movements within the volcano plumbing system^1,15^. The visual inspection of such a baseline allows detecting three main ground deformation stages, passing from no significant changes (May 19, 2016–June 22, 2016; hereinafter T1) to a period characterized by a baseline lengthening of ~4 cm (June 22, 2016–March 15, 2017; hereinafter T2), followed by a period characterized by a baseline shortening of 1 cm (March 15, 2017–April 1, 2017; hereinafter T3). The baseline started to length again after April 1, 2017, remarking the onset of a new inflation of the volcano edifice, which culminated with a dike intrusion feeding a vigorous Strombolian activity and lava flow effusion on late December 2018.

**Figure 5:**
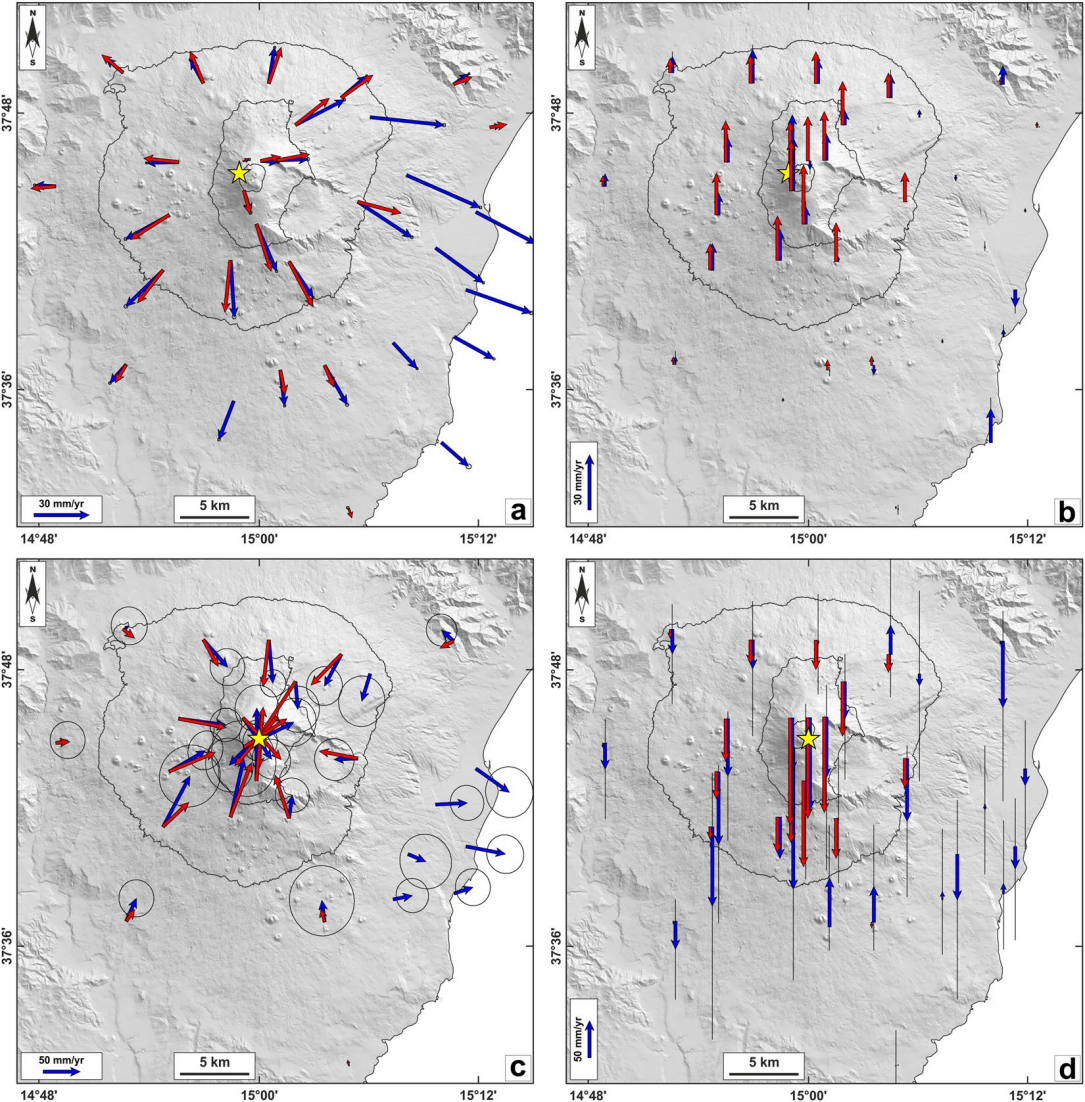
Comparison between observed (blue arrows) and modeled (red arrows) horizontal (**a,c**) and vertical (**b,d**) ground deformation fields relevant to the considered time intervals: (**a,b**) for T2 (June 22, 2016–March 14, 2017) and (**c,d**) for T3 (March 14–April 1, 2017). The surface projections of modeled sources are reported as yellow stars. Parameters of the modeled sources are reported in Table 1. Stations located on the middle-to-lower eastern flank of the volcano have been discarded from modeling because they are affected by long-term seaward motion^32^. T1 period (May 19, 2016–June 22, 2016) showed no significant deformation field, therefore it was not reported.

Some differences in the general lengthening pattern can be recognized during T2, namely: (a) a fast lengthening (~1 cm) from June 22, 2016 to July 2, 2016 (T2a in Fig. 4); (b) a prolonged period of almost constant lengthening from July 2, 2016 to February 28, 2017 (T2b in Fig. 4); (c) a null to very small lengthening from February 28, 2017 to March 15, 2017 (T2c in Fig. 4). It is also worth to note a period of null deformation during T2b observable from January 28, 2017 to February 9, 2017, which is well related to the ash emission accompanying the weak explosive activity occurred at the vent located on the SEC-NSEC saddle in late January 2017. Other differences can be observed in the general shortening pattern during T3, namely: (a) a fast shortening (−0.7 cm) from March 15, 2017 to March 21, 2017 (T3a in Fig. 4); (b) a slow pattern of shortening (−0.3 cm) from March 21, 2017 to April 1, 2017 (T3b in Fig. 4). Because of the low signal/noise ratio characterizing these sub-periods, we refer only to the general T2 and T3 patterns in the following computations. Therefore, by combining the daily GAMIT solutions into a consistent set of station positions and velocities and adopting a local reference frame to isolate the volcanic deformation from the background tectonic pattern^16^, we estimated the ground deformation field (in terms of geodetic velocities) for T2 and T3 stages (Fig. 5).

The surface deformation for both detected stages was used to constrain isotropic half-space elastic inversion models by using a genetic algorithms approach^17^ and taking into account the effects of topography^18^. The assumption of purely elastic deformation approximation could be inappropriate due to the typical presence of high temperature gradients in volcanic areas (hundreds of degrees per kilometer) for which the medium is likely to behave inelastically over long time scales. However, our choice to consider an isotropic and elastic medium, common to most works on volcanic deformation modeling^13^, is justified because small displacement gradients are usually observed^19^. Furthermore, the fairly good fit between observed and modeled ground deformation indicates that the behavior of the volcano at the timescales analyzed in this study can be considered compatible with an elastic behavior.

We adopted a finite prolate spheroid source^20^ to model the observed ground deformation patterns. Values of 30 GPa and 0.25 were used^13,14^ for the shear modulus (*μ*) and Poisson’s ratio in the half-space, respectively. Estimation of the uncertainties in best fitting parameters was performed by adopting a Jackknife sampling method^21^. In the computation, both horizontal and vertical GPS components were inverted by taking into account the weights proportional to the estimated geodetic velocity errors. During the inversions, in order to obtain realistic estimations of the pressure change parameter, we limited the ratio of the pressure change to the shear modulus within the elastic limits of the surrounding rock^22^. The volume change *∆V* of the ellipsoidal cavity can be approximated by the following empirical formulation^23^:

$${\rm{\Delta }}V=\frac{3V{\rm{\Delta }}P}{4\mu }(\frac{{A}^{2}}{3}-cA+d)$$

where *V* is the volume of the ellipsoidal cavity, *μ* is the rocks shear modulus, *∆P* is the pressure change, *A* = *b/a* represents the geometric aspect ratio between the semi-major axis *a* and the semi-minor axis *b* of the prolate spheroid source^20^. Constants *c* and *d* are determined by the polynomial best fit to the numerical solutions^24^ and have values of 0.7 and 1.37, respectively. Parameters of the modeled sources inferred for T2 and T3 stages are reported in Table 1, whereas the modeled ground deformation patterns are reported in Fig. 5.

**Table 1:** Parameters of the modeled sources inferred for the deformative stages analyzed in this study.

Parameters	T2	T3
Easting (m)	498517 ± 187	500111 ± 456
Northing (m)	4178975 ± 168	4178250 ± 516
Depth (m bsl)	6298 ± 350	4640 ± 434
a (m)	1594 ± 77	438 ± 43
b/a ratio	0.25 ± 0.01	0.68 ± 0.03
Azimuth (°)	211 ± 11	227 ± 3
Dip (°)	79 ± 5	1 ± 10
ΔP (Pa)	3.7 ± 0.3 · 10^8^	−3.2 ± 0.2 · 10^8^
ΔV (10^6^ m^3^)	11.8 ± 4.0	−1.38 ± 0.6

## Discussion

Bulk composition of volcanic rocks erupted during the February–April 2017 eruptions have been considered within the framework of the post-2011 activity at Mt. Etna. All volcanic rocks are K-trachybasalts with major element compositions comparable to those of other recently erupted products, with particular reference to those emitted at VOR during 2015–2016^6,10,11^. Major and trace element data suggest that magmas feeding the 2017 eruptions generally preserve the same geochemical signature with respect to those feeding the violent paroxysmal eruptions of December 2015 and May 2016 at VOR. Indeed, the rather constant incompatible trace element ratios of the VOR 2015–2016 and the 2017 products are in accordance with an evolutionary path principally controlled by a crystal fractionation process along a very similar liquid line of descent (Fig. 2). The slight differences between the VOR 2015–2016 and 2017 products observed especially for the HFSE group seem to be related to higher proportions of augitic clinopyroxene in the VOR 2015–2016 products, a feature that could have been acquired through processes of selective accumulation during magma storage at depth.

We have combined our whole rock data with information preserved in olivine crystals to explore dynamics of magma ascent and interaction leading to the activity of 2017. Olivine cores spanning from more basic (Fo_80–81_) to slightly evolved compositions (Fo_72–74_) reflect crystal growth in separated magma volumes residing at different depths beneath Mt. Etna, each defined by precise physical and chemical conditions. Specifically, the four olivine populations that we have identified within the 2017 eruptive products fall in the same range of compositions of olivine groups recognized by several authors for past effusive and explosive eruptions of Mt. Etna^7,10,11,25,26^. Such observations lead us to infer that the residence and growth of olivine crystals may have happened within the same magmatic environments (indicated as M_i_) that were selectively reactivated during the 2011–2013 eruptive sequence at the NSEC, during the July–August 2014 eruption at the NEC-NSEC and later between 2015 and 2016 during the volcanic activity at VOR^7,10,11^. The thermodynamic MELTS simulations available for the 2011–2013 and 2015–2016 volcanic products^10,11^ have been therefore used to fix the X-P-T crystallization conditions of the 2017 magmas. Following this thermodynamic modeling, Fo_80–81_ olivine cores are representative of the M_0_ environment located at pressure of 420–380 MPa. Fo_78_ cores refer to the M_1a_ magma environment at 290–230 MPa, whereas olivines with Fo_76_ core compositions belong to the M_1b_ environment at 160–120 MPa. The Fo_72–74_ core compositions encompass a different compositional range if compared with the previously individuated olivine populations^7,10,11^. Olivines with Fo_74_ core compositions are the most frequent within this population. On the whole, this potentially new magmatic environment allowing the Fo_74_ core crystallization can be considered intermediate between that of M_1b_ magma (with olivine cores at Fo_75–76_) and the composition of a more evolved magma residing at 30–40 MPa with Fo_70–72_ cores under equilibrium (i.e., the M_2_ magmatic environment)^10^. Specifically, on the basis of the thermodynamic parameters, the Fo_74_ cores would have crystallized at pressure of 70–90 MPa and temperatures between 1094–1098 C° ^10^. In any case, the olivine core population at Fo_72–74_ found in the 2017 lava testifies a stage of crystallization in the shallowest levels of the plumbing system into a M_2_-like magmatic environment.

**Figure 6:**
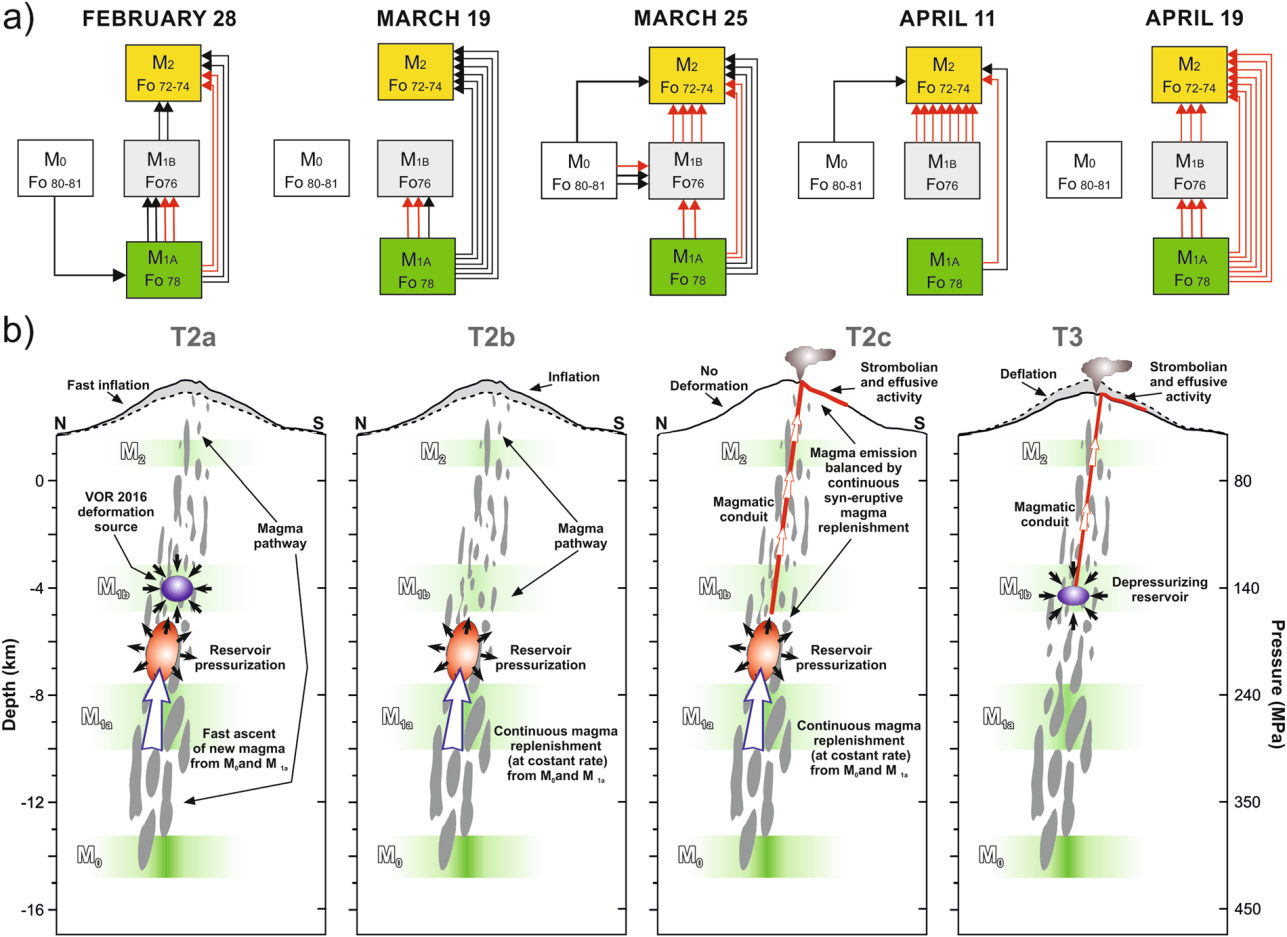
(**a**) Connections between the magmatic environments defined through the zoning patterns of olivine crystals for the eruptive episodes of 2017. Each colored box represents a different magmatic environment. Arrows indicate the direction of magma transfer as recorded from olivine zoning. The number of arrows indicates the number of connections identified from the crystal zoning (normal or reverse); black arrows: normal zoning; red arrows: reverse zoning patterns. (**b**) Schematic cartoon of magma movements and deformation sources within the Mt. Etna plumbing system between June 2016 (i.e., at the end of the volcanic activity at VOR) and April 2017. The inflation sources (in red) are reported for the panels relative to T2a, T2b and T2c. The deflation sources (in blue) are reported in panels T2a (which is related to that of the May 2016 eruption at VOR) and in T3 for the 2017 eruptive activity. Magmatic environments have been also reported in the range of pressures defined from thermodynamic modeling available in literature^10^, whereas depth ranges have been constrained on the basis of the density model calculated for the basement underlying Mt. Etna^33^.

Considerations on the core composition with various zoning patterns of the four olivine groups allow to define routes of connections between such magmatic environments, as well as processes of recharge and mixing preceding each eruptive episode over the period under investigation (Fig. 6a). The chemical analysis of olivine crystals reveals that, in spite of the complex diversity of zoning patterns, some evolutionary paths are dominant in the history recorded by each population. For instance, the prevalence of normal zoning pattern characterizing olivines with core composition Fo_80–81_ and Fo_78_ for the February 28 and March 19 eruptions clearly indicates ascent of magmas from the deep reservoirs (M_0_ and M_1a_) to shallow crustal levels. Over these first months of volcanic activity, the transfer of the M_0_ and M_1a_ magmas throughout the intermediate reservoir M_1b_ up to M_2_ is tracked by the slightly reverse zoning patterns of the Fo_76_ olivines (maximum Fo_78_). Since March 25, olivine cores with Fo_72–74_ start to increase (with prevalence of Fo_74_ cores), being also evidence for the reactivation of the shallow magmatic environment M_2_-like. Marked reverse zoning surrounding the Fo_76_ to Fo_72_ cores, which record Fo increase to Fo_76–81_, confirms the occurrence of subsequent episodes of recharge by magmas coming from M_0_ and M_1a_ that involved the shallow (<160 MPa) portion of the plumbing system.

Petrological evidence of multiple magmatic environments beneath the volcano appears strongly connected with results coming from the modeling of geodetic data, which infer the presence of two active magmatic sources associated to the T2 and T3 ground deformation stages. For the deformation stage T2 (from June 16, 2016 to March 15, 2017; Fig. 5a,b), the modeled magmatic source is located beneath the western side of the summit area at depth of ~6.3 km bsl. Such a source is given by a near vertical prolate spheroid and is characterized by a positive volume change of 11.8 × 10^6^ m^3^. For the T3 stage (from March 16 to April 1, 2017; Fig. 5c,d), the modeled source is located beneath the summit area at depth of ~4.6 km bsl. Such a source is given by an oblate spheroid and is characterized by a negative volume change of 1.4 × 10^6^ m^3^. Based on the estimated depths and associated uncertainties, the modeled sources do not overlap, and therefore they represent two distinct portions (or magmatic reservoirs) of the volcano plumbing system. The source inferred for T3 at ~4.6 km bsl well overlaps, within the estimated uncertainties, with the sources inferred for the inflation and deflation deformative stages related to the 2015–2016 volcanic events at VOR, covering the same pressure range of the M_1b_ environment olivines with Fo_76_ cores^11^. Differently, the source inferred for T2 is ~1.7 km deeper than that of T3, i.e. vertically placed between the M_1a_ and M_1b_ environments (see also Fig. 6b).

**Figure 7:**
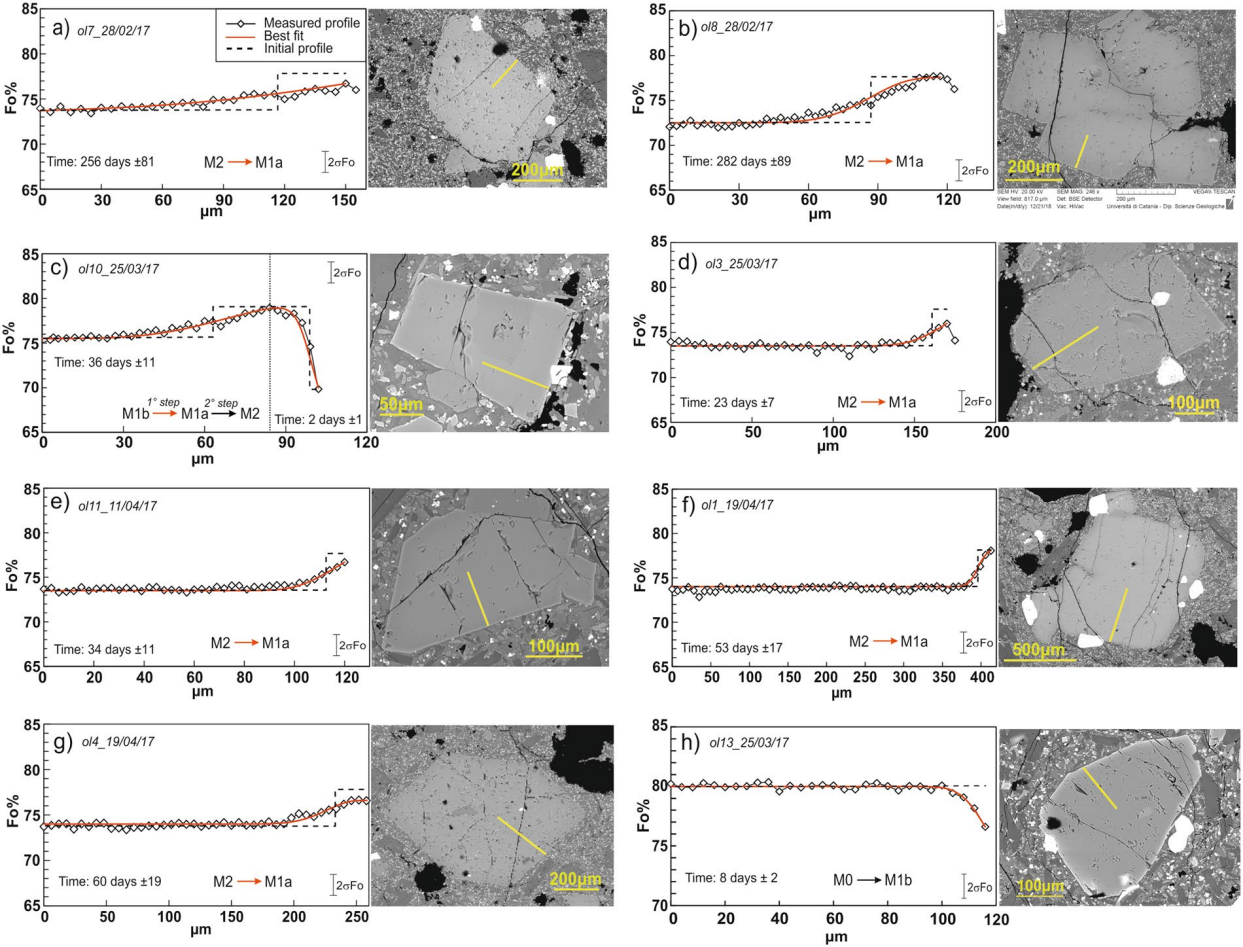
Fe-Mg diffusion modeling in representative olivine crystals found in February–April 2017 volcanic products. Calculated timescales with relative uncertainties are reported for each step of the modeling. Red lines represent the best fit for diffusion curves, whereas black dashed lines indicate the initial concentration profile prior to the diffusion. Yellow lines in the BSE images indicate the direction of the SEM-EDS/WDS compositional traverses.

Although located at different depths, the geodetically inferred magmatic sources are connected and they strictly interacted before and during the eruptive events of 2017. As such processes of magma movement and interaction are also well evidenced by the compositional heterogeneities in olivine crystals, we were able to recover the timescales associated to these processes through modeling based on the diffusional smoothing of the Fe-Mg zoning of olivine crystals. The modeling strategy and criteria of selection of the crystals used for time determinations are consistent with those adopted for the investigation of pre-eruptive dynamics at Mt. Etna during the period 2011–2013, 2014 and 2015–2016^7,10,11^. Timescales determined for all modeled olivine crystals, along with the thermodynamic parameters used for diffusion calculations are summarized in Table 2. Final results show a reasonable fit between the observed deformation patterns (and associated models) and the inferred transfer dynamics from olivine zoning, allowing to follow the evolution through time of the volcanic activity between February and April 2017 (Fig. 4). Specifically, a few days after the end of the May 2016 volcanic events at VOR, ground deformation data record a new inflation of the volcano edifice, which lasted about 10 months and is marked by the T2 period (Fig. 4). The ascent of new magmas producing the inflation stage T2 is tracked by the reverse zoning of Fo_72–74_ and Fo_76_ crystals erupted on February 28. The diffusive relaxation of such zoning patterns produces timescales of about 209–282 days (±64–89 days; Fig. 7a,b; Table 2), which are consistent with processes of magma movement that started 8–10 months before the beginning of the 2017 eruptive activity, i.e. at the end of the May 2016 eruptions occurred at VOR. Some differences occur in the deformation rate of the T2 period, especially on its initial and final periods as evidenced by T2a and T2c in Fig. 4. Indeed, the early period T2a from June 22, 2016 to July 2, 2016 shows a deformation rate that was twice with respect to that of T2b from July 2, 2016 to February 28, 2017. Conversely, a very low deformation rate characterizes the period T2c from February 28, 2017 to March 15, 2017. We suggest that during T2a, the fast ascent of magma from depth was triggered by the pressure imbalance between the magmatic environment M_1b_ and more deep environments, which could be well represented by M_1a_ and/or M_0_. Such a pressure imbalance was related to the sudden unloading of the magma column at the M_1b_ level because of the violent paroxysmal activity at VOR in May 2016. This inference finds support considering the deformation picture preceding the 2017 eruptive episodes. Indeed, the depth of the deflation source related to the eruptive events of May 2016 at VOR well coincides with the M_1b_ environment, whereas pressurization of the source leading to the T2 inflationary period lies about 2 km below M_1b_ (Fig. 6).

**Table 2:** Parameters used for Fe-Mg diffusion modeling on olivine crystals with estimation of timescales and their relative uncertainties.

Date	Crystal	T (°C)	P (MPa)	*f*O_2_ (bar)	Mi	Δt ± σ
*Feb-28*	*ol4_28/02/17*	1108	140	10^−9.28^	M1b-M1a *(r)*	209 ± 64
	*ol7_28/02/17*	1084	30	10^−9.65^	M2-M1a *(r)*	256 ± 81
	*ol8_28/02/17*	1084	30	10^−9.65^	M2-M1a *(r)*	282 ± 89
*Mar-19*	*ol3_19/03/17*	1131	260	10^−8.92^	M1a-M2 *(n)*	14 ± 4
	*ol12_19/03/17*	1108	140	10^−9.28^	M1b-M1a *(r)*	98 ± 30
*Mar-25*	*ol1_25/03/17*	1108	140	10^−9.28^	M1b-M1a *(r)*	30 ± 9
		1108	140	10^−9.28^	M1b-M2 *(n)*	5 ± 2
	*ol3_25/03/17*	1084	30	10^−9.65^	M2-M1a *(r)*	23 ± 7
	*ol4_25/03/17*	1084	30	10^−9.65^	M2-M1a *(r)*	44 ± 14
	*ol6_25/03/17*	1084	30	10^−9.65^	M2-M1a *(r)*	34 ± 11
	*ol10_25/03/17*	1108	140	10^−9.28^	M1b-M1a *(r)*	36 ± 11
		1108	140	10^−9.28^	M1a-M2 *(n)*	2 ± 1
	*ol12_25/03/17*	1108	140	10^−9.28^	M1b-M1a *(r)*	34 ± 10
	*ol13_25/03/17*	1164	390	10^−8.49^	M0-M1b *(n)*	8 ± 2
*Apr-11*	*ol1_11/04/17*	1084	30	10^−9.65^	M2-M1a *(r)*	34 ± 12
	*ol2_11/04/17*	1084	30	10^−9.65^	M2-M1a *(r)*	26 ± 8
		1084	30	10^−9.65^	M1a-M2 *(n)*	14 ± 4
	*ol4_11/04/17*	1084	30	10^−9.65^	M2-M1a *(r)*	38 ± 12
	*ol5_11/04/17*	1164	390	10^−8.49^	M0-M1a *(n)*	8 ± 2
	*ol7_11/04/17*	1084	30	10^−9.65^	M2-M1a *(r)*	30 ± 10
	*ol9_11/04/17*	1084	30	10^−9.65^	M2-M1a *(r)*	24 ± 8
		1084	30	10^−9.65^	M2-M1a *(r)*	11 ± 4
	*ol10_11/04/17*	1084	30	10^−9.65^	M2-M1a *(r)*	34 ± 7
	*ol11_11/04/17*	1084	30	10^−9.65^	M2-M1a *(r)*	34 ± 11
*Apr-19*	*ol1_19/04/17*	1084	30	10^−9.65^	M2-M1a *(r)*	53 ± 17
	*ol2_19/04/17*	1084	30	10^−9.65^	M2-M1a *(r)*	45 ± 14
	*ol3_19/04/17*	1108	140	10^−9.28^	M1b-M1a *(r)*	45 ± 14
	*ol4_19/04/17*	1084	30	10^−9.65^	M2-M1a *(r)*	60 ± 19
	*ol5_19/04/17*	1108	140	10^−9.28^	M1b-M1a *(r)*	43 ± 16
		1108	140	10^−9.28^	M1a-M2 *(n)*	8 ± 2
	*ol7_19/04/17*	1084	30	10^−9.65^	M2-M1a *(r)*	48 ± 15
	*ol8_19/04/17*	1084	30	10^−9.65^	M2-M1a *(r)*	30 ± 10
	*ol9_19/04/17*	1084	30	10^−9.65^	M2-M1a *(r)*	48 ± 15
	*ol10_19/04/17*	1108	140	10^−9.28^	M1b-M1a *(r)*	39 ± 12
	*ol11_19/04/17*	1108	140	10^−9.28^	M1b-M1a *(r)*	30 ± 9
	*ol12_19/04/17*	1084	30	10^−9.65^	M2-M1a *(r)*	34 ± 11
	*ol13_19/04/17*	1108	140	10^−9.28^	M1b-M1a *(r)*	55 ± 17

Δt refers to the time of diffusion calculated for a single zoning type, i.e., normal zoning (*n*) or reverse zoning (*r*). Note that crystals characterized by complex zoning patterns have been modeled in multiple steps, and Δt is therefore defined for each step modeling.

During the T2b period, the plumbing system of the volcano pressurized at a constant rate until late February. The beginning of the eruptive activity at the end of February 2017 was likely driven by injection of new magmas into the very shallow portions of the volcano plumbing system, corresponding to the M2 environment. This process is well evidenced by the reverse zoning of olivine crystals erupted on March 25, which record increasing Fo concentrations from Fo_72–74_ to Fo_76–78,_ providing evidence for the M2 reactivation as a consequence of magma replenishment from the M_1a_ and M_1b_ environments. Based on time calculations, this magma injection occurred 23–44 days (±7–14 days; Fig. 7c,d; Table 2) before March 25 (i.e., end of February). Olivines with reverse zoning erupted between April 11 and April 19 also record episodes of recharge from the M_1a_ and the M_1b_ environments with diffusion timescales of 30–60 days (±10–19; Fig. 7e–g; Table 2), dating back to the T2c period (from February 28 to March 15, 2017). These data, coupled with the almost null deformation observed during the T2c period, indicate that the recharge was almost continuous and has balanced the magma discharge at the surface.

On March 16, 2017 effusive activity occurred along the fracture opened at the base of SEC, therefore leading to the contemporaneous deflation of the volcano (T3 in Fig. 6). The deflation occurred at fast rates during the first week (T3a in Fig. 6), then progressively diminished from March 21 to April 1 (T3b in Fig. 6) due to decrease of the effusive activity from the fracture. Although a timescale of 8 days (±2; Fig. 7h; Table 2) has been obtained only for a few normally-zoned Fo_80–81_ olivines in lava rocks of March 25 and April 11, this result could suggest that the most basic M_0_ magma rapidly moved upward to the surface during T3.

## Conclusions

All the petrological and geodetic data support a model of self-feeding magma replenishment originated as a consequence of the violent paroxysmal eruptions occurred at VOR in May 2016. The extraordinary volcanic phenomena at VOR may have also caused a radical change of the volcano plumbing system dynamics, especially for what concerns the capability and efficiency to transfer magma from the deep levels of the storage zones upward to the surface. On the basis of the available data, in this studied case, the best plumbing system configuration should be a sort of nearly continuous magma column with steady state behavior, where each magma output at the surface was compensated by magma addition at depth. Support to this inference is given by the nearly flat deformative pattern observed during T2c, which corresponds to the beginning of magma emission during the early eruptive episodes of February–March 2017 (Fig. 6). Indeed, the deflation expected as a consequence of magma extrusion did not occur, probably due to the continuous magma injections from depth that balanced effusive phenomena at the surface. Efficiency of the self-feeding process of magma recharge decreased since March 16, when the explosive activity and fracture opening at the base of SEC caused more efficient magma drainage, which probably perturbed the steady state behavior. Reverse zoning and timescales obtained in olivine crystals, together with the beginning of an inflationary deformative trend after T3, support the idea that the steady state with consequent self-feeding magma replenishment into the plumbing system was restored at least since April 1. Indeed, magma discharges occurred during the April 11, 19 and 27 eruptions were anomalously accompanied by a continuous inflationary deformative pattern.

Petrological and geodetic data presented in this study provided useful constraints on magma movements along the plumbing system of the volcano during the entire investigated time interval, providing a sounding reconstruction of the spatial-temporal history of magmas involved into the February–April 2017 eruptive events. Such a petrological-geodetic integrated analysis affords a powerful tool to gain insights into complex modes of magma supply that could be hardly recognized by the collection and interpretation of data coming only from a single discipline.

## Methods

### Geochemical analyses

Major element compositions for all the collected samples were analyzed at the Dipartimento di Biologia, Ecologia e Scienze della Terra of the University of Calabria (Cosenza, Italy) by means of a Philips PW2404 WD-XRF on powder pellets correcting the matrix effects; loss on ignition was determined by gravimetric methods. Together with the 2017 volcanic rocks, we have also re-analyzed in the same laboratory major elements of the 2015–2016 products erupted at VOR in order to have a dataset not affected by analytical bias. Trace element abundances on the 2017 volcanic rocks were measured at the SGS Laboratories of Lakefield (Ontario, Canada). Powdered rock samples were fused by Na-peroxide in graphite crucibles and dissolved using dilute HNO_3_. Elemental analyses were then obtained by means of a Perkin Elmer ELAN 6100 inductively coupled plasma mass spectrometer. Four calibration runs were performed on international certified reference materials (USGS GXR-1, GXR-2, GXR-4 and GXR-6) at the beginning and end of each batch of 5 samples. Precision is better than 3–5% depending on the analyzed element, accuracy is on the same order of magnitude. The complete dataset for the analyzed samples is available as Supplementary Table S3.

Sixty-nine olivine crystals were selected for *in situ* micro-analyses along core-to-rim transects, with spacing between each spot analysis on the order of 3–7 µm. All the high-contrast back-scattered electron images (BSE; 1024 × 864 pixels) and major element compositions on olivine crystals were obtained at the Dipartimento di Scienze Biologiche, Geologiche e Ambientali of the University of Catania (Italy) by means of a Tescan Vega-LMU scanning electron microscope equipped with an EDAX Neptune XM4–60 EDS micro-analyzer characterized by an ultra-thin Be window coupled with an EDAX WDS LEXS (wavelength dispersive low energy X-ray spectrometer) calibrated for light elements. Operating conditions were set at 20 kV accelerating voltage and ~8 nA beam current for obtaining high-contrast BSE images and 20 kV accelerating voltage and ~2 nA beam current for the analysis of major element abundances in olivines and the other mineral phases for their compositional characterization. Repeated analyses on internationally certified standards (olivine and glass) during the analytical runs ensure precision for all the collected elements on the order of 3–5%, whereas accuracy is on the order of 5%.

### Modeling of chemical zoning in olivine crystals

The diffusive relaxation of olivine chemical zoning patterns follows the Fick’s second law. Compositional variations of olivine crystals are described as a function of space (x) and time (t) and are related to values of the diffusion coefficients (D) along the measured profile. Here, we solved the diffusion equation numerically by using the method of finite difference^27,28^. Starting from the initial concentration profile of the crystal, diffusion curves may be tracked for multiple temporal steps, until a new profile that fits the observed concentration profile is obtained (see the Supplementary Table S4). The duration of diffusion in some crystals was modeled at different stage of crystallization before eruption, and the crystal boundaries were considered to be open. Diffusion in olivine is highly anisotropic, therefore crystals were preliminarily selected to minimize uncertainties on time determinations related to the section orientation with respect to the fast diffusion direction (c axis)^29,30^. The modeling has been performed by using concentration-dependent diffusion coefficients (D_Fe-Mg_) that were calculated along [001]^31^. As diffusion coefficients depend on the mole fraction of the fayalite component (X_Fe_), oxygen fugacity (ƒO_2_), pressure (P), temperature (T), we calculated different coefficients for each identified olivine population by using the T, P and ƒO_2_ parameters that define a physical environment consistent with the range of crystallization conditions recognized at Mt. Etna for the post-2011 activity and that were determined through the rhyolite-MELTS simulations (see Supplementary Table S4; Table 2)^10^. The diffusion coefficient for a given profile direction (Dtrav) can be calculated when the orientation of the chemical profile relative to the crystallographic axes of the olivine is known^29,30^. The orientation of the olivine crystallographic a, b and c axes (respectively coincide with the optical indicatrix axes Z, X and Y) were measured by conoscopic observations under a polarizing optical microscope equipped with a Zeiss 4 axis universal stage at the University of Catania. Measurements of the anisotropy-corrected diffusivity along the direction of the profile further improve the accuracy and precision on time calculations. Despite the number of crystals suitable for diffusion calculation is reduced to 32 olivine crystals after application of all the selection and correction criteria (i.e., influence of sectioning, diffusion anisotropy, crystal morphology)^29,30^, the adopted modeling approach has revealed a high degree of robustness as the timescales obtained from multiple crystals that experienced the same evolutionary history are rather consistent with each other.

Uncertainties on timescales determination were calculated by propagating the error in the determination of diffusion coefficient *D*. We used standard procedures for error propagation in the Arrhenius expression in order to determine the uncertainty on *D*, which is primarily governed by changes in temperature and oxygen fugacity^26^. The error propagation analysis was, therefore, performed for each olivine population fixing σ_*T*_ = 10 °C and σ_*logfO2*_ = 0.25, respectively. Under these conditions, calculated uncertainties range from ±1 days for the shorter timescales to ±89 for the longer timescales (Table 2).

## Supplementary information

Table S1

Table S2

Table S3

Table S4
